# Case report: Atypical Parkinsonism following SARS-CoV-2 infection

**DOI:** 10.3389/fneur.2023.1208213

**Published:** 2023-07-31

**Authors:** Paola Polverino, Tiziana De Santis, Elena Perdixi, Adriano Chiò, Alberto Albanese

**Affiliations:** ^1^Department of Neurology, IRCCS Humanitas Research Hospital, Milan, Italy; ^2^ALS Centre “Rita Levi Montalcini,” Department of Neuroscience, University of Turin, Turin, Italy; ^3^Azienda Ospedaliero-Universitaria Città della Salute e della Scienza, Turin, Italy; ^4^Department of Neuroscience, Catholic University, Rome, Italy

**Keywords:** COVID-19, multiple system atrophy, Parkinsonism, SARS-CoV-2, diagnosis

## Abstract

A wide range of neurological manifestations have been reported during the COVID-19 pandemic, including a variety of Parkinsonian cases. The association of numerous viruses with the development of persistent or transient Parkinsonism has been well-documented. We observed a patient who developed a levodopa non-responsive Parkinsonian syndrome with dysautonomia during a prolonged stay at home for COVID-19. Although the temporal proximity of the emerging Parkinsonian features with a COVID-19 diagnosis suggested a causal relationship, we considered the possibility of a coincidental occurrence of multiple system atrophy. We discuss the patient's clinical features in relation to the established clinical diagnostic criteria and review differential diagnoses as well as the role of SARS-CoV-2 infection.

## Introduction

Parkinsonian syndromes include idiopathic Parkinson's disease (PD), progressive supranuclear palsy, multiple system atrophy (MSA), corticobasal degeneration, and vascular Parkinsonism, among other rarer causes of Parkinsonism. Etiology is considered multifactorial, resulting from the contribution of environmental, genetic, and epigenetic factors. Viruses are recognized environmental causes of Parkinsonism, including influenza A, Epstein–Barr virus, hepatitis C virus, varicella zoster, West Nile virus, and Japanese encephalitis virus (1). The number of cases of COVID-19-related Parkinsonism have been described during the recent pandemic outbreak and linked to the premorbid infection (2). We observed a patient presenting with Parkinsonism and dysautonomia following a prolonged SARS-CoV-2 infection.

## Setting and methods

Assessments were performed at the Department of Neurology, Humanitas Research Hospital (Rozzano, Milan, Italy). Patient clinical data were stored in the hospital's electronic medical records. Clinical and laboratory procedures were performed according to hospital protocols and good clinical practice guidelines. The case description conforms to CARE guidelines (3). Written informed consent was obtained from the participant for the publication of this case report, including clinical data and images.

## Case presentation

A 62-year-old right-handed man, working as a swimming pool manager, received emergency admission in March 2020 because of fever and mild respiratory symptoms. Severe osteoporosis and bilateral glaucoma were reported in his medical records. He did not present hyposmia or sleep disorders. Family history was unremarkable for Parkinsonism or other neurological conditions. A nasopharyngeal swab tested positive for SARS-CoV-2, but chest CT did not show pneumonia. Having mild COVID-19 symptoms, he was treated at home with paracetamol and low-molecular-weight heparin. Respiratory symptoms recovered in approximately 20 days; in total, he remained isolated at home for approximately 3 months until a nasopharyngeal swab tested negative. During the last few weeks of isolation at home, his family members noticed abnormal cervical posturing associated with ideomotor slowing and progressive gait instability, causing a fall and rib fractures. Neck dystonia gradually progressed, and global bradykinesia became apparent. The patient received dopamine replacement therapy (up to 150 mg daily of levodopa with benserazide) that yielded no appreciable motor improvement.

**Figure 1:**
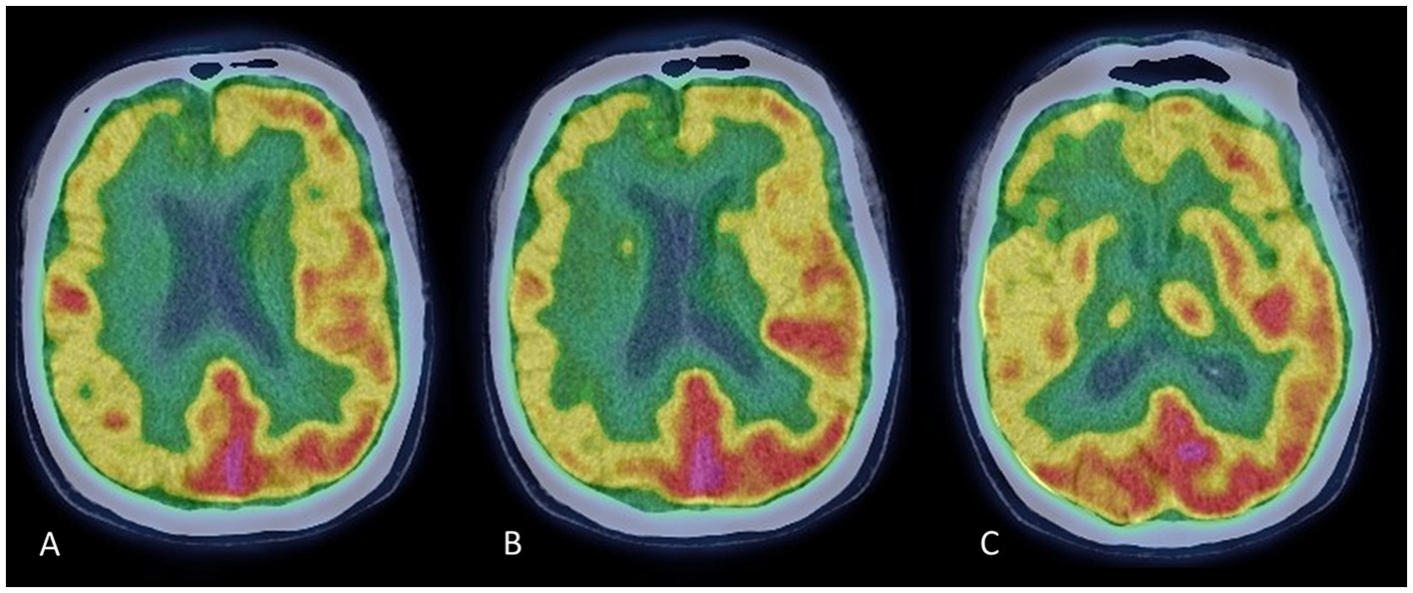
Brain FDG-PET scans at three rostrocaudal levels **(A–C)**. Images show a pattern of diffuse hypometabolism involving the right frontal and frontotemporal cortex, which is more evident in the medial regions.

In March 2021, a neurological examination showed bilateral rigid-akinetic Parkinsonism, with slight prevalence on the left-hand side. There was axial involvement with mild postural instability and neck dystonic posturing. The MDS-UPDRS motor score was 18/132, and the Hoehn and Yahr stage was 3. There were no cerebellar or pyramidal signs and no complaints of orthostatic hypotension. Dopamine replacement therapy was increased up to 450 mg daily (t.i.d.) of levodopa with benserazide, and a rotigotine patch was administered at a dose of 4 mg per day. These medications were well tolerated but provided no significant motor improvement. Brain MRI was unremarkable, and single-photon emission computed tomography (SPECT) with ^123^I-Ioflupane showed a marked bilateral reduction in presynaptic dopaminergic binding. Brain ^18^F-FDG-PET revealed right frontal and frontotemporal hypometabolism, especially in the medial regions (Figure 1). Neuropsychological assessment revealed mild long-term memory difficulties for visuospatial material, slight attentive and executive dysfunction, and apathy. Autonomic testing revealed mild sympathetic autonomic dysfunction. The SCOPA-AUT score was 11/69. An extensive whole-exome NGS test showed no variants in genes related to Parkinsonism or other movement disorders.

**Figure 2:**
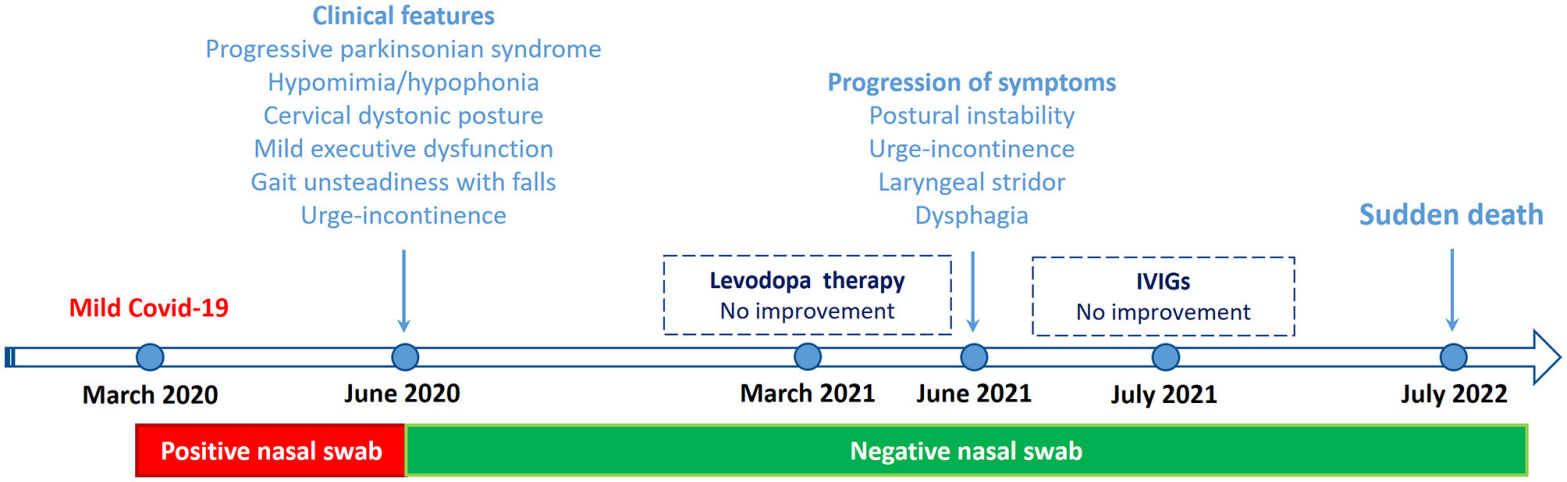
Clinical course and key events from March 2020 to July 2022. IVIGs, Intravenous immunoglobulins.

The patient's picture quickly worsened. In June 2021, gait became unsteady with frequent falls; there were initial dysphagia and inspiratory stridor. Urinary symptoms progressively worsened, with urge incontinence, increased urinary frequency, and incomplete bladder emptying (SCOPA-AUT score: 17/69). In July 2021, the patient was taking 450 mg of levodopa with benserazide and 4 mg rotigotine, still with no evidence of efficacy. The MDS-UPDRS motor score was 24/132. Rotigotine was withdrawn, and levodopa medication was maintained. In August 2021, intravenous immunoglobulins were administered (0.4 g/kg/daily, total dose 30 g) without appreciable benefit. The patient's motor condition continued to worsen: inspiratory stridor was reported, gait impairment and dysphagia became prominent, and urinary dysautonomia progressed. The Hoehn and Yahr stage was 4 (Figure 2).

In July 2022, the patient died suddenly at home while sleeping. He was found dead in the morning. Disease duration was reckoned to have lasted ~2 years from motor onset. The family did not agree on performing an autopsy.

## Discussion

The patient in this vignette presented with a non-tremulous Parkinsonian syndrome characterized by rigidity, bradykinesia, dystonia, and gait impairment, associated with dysautonomia. Atypical Parkinsonian features included a lack of response to dopaminergic medication, early cervical dystonia, and early postural instability with falls. The differential diagnosis is reported in Box 1.

Box 1 **Differential diagnosis of the reported patient.**

1. COVID-19-related Parkinsonism. The main cue to this diagnosis is the close chronological relation between SARS-CoV-2 infection and the onset of motor Parkinsonian features. However, no additional evidence for a causal relationship was collected. The patient lacked clinical, laboratory, or neuroimaging findings of encephalopathy. Reduced presynaptic dopamine transporter uptake suggested a pre-existing neurodegenerative process involving the basal ganglia.
2. Other coincidental neurodegenerative Parkinsonism. The clinical presentation did not match the current clinical diagnostic criteria for PD (11). Rapid progression of gate impairment and recurrent falls within 3 years of onset were red flags, while the absence of observable improvement with dopamine replacing therapy was an absolute exclusion criterion. The criteria for diagnosis of corticobasal degeneration were also not fulfilled (17). The diagnosis of progressive supranuclear palsy (PSP) was consistent with a history of frequent falls but unsupported by other core clinical features and excluded by the occurrence of autonomic dysfunction (18).
3. mmune-mediated atypical Parkinsonism associated with dysautonomia has been reported. In these cases, however, Parkinsonian features are outweighed by cerebellar and hyperkinetic features; seizures and other neurological manifestations are also found (19). The patient had only Parkinsonism with dysautonomia; treatment with immunoglobulins was empirically tested without efficacy. High-dose steroids were not administered because of severe osteoporosis. A second cycle of immunoglobulins or plasmapheresis was not justified in this case.
4. Coincidental paraneoplastic syndrome. Isolated cases of paraneoplastic Parkinsonism with atypical presentation have been reported. One patient with anti-CV2 antibody manifested Parkinsonism and autonomic dysfunction with normal neuroimaging, suggesting a diagnosis of multiple system atrophy (20). This rather unique presentation benefitted from immunotherapy. Other Parkinsonian cases associated with anti-CRMP5 antibodies had no dysautonomia and additionally displayed bilateral signal hyperintensities in the basal ganglia. Rare presentations of anti-Ri and anti-Ma2 antibodies include Parkinsonism with supranuclear gaze palsy, usually associated with brainstem or cerebellar dysfunction, and oculomotor abnormalities (21): these features were not observed in this case. In these cases, MRI abnormalities are typically observed (12). Although this does not exclude with certainty a paraneoplastic cause, the patient had no evidence of malignancies, imaging was unremarkable, and no appreciable benefit from immunoglobulin treatment was assessed.

This patient met the diagnostic criteria for clinically probable MSA (4). The core diagnostic features were autonomic dysfunction, consisting of urinary urge incontinence, and Parkinsonism. The supportive diagnostic features were rapid progression and postural instability within 3 years of motor onset, craniocervical dystonia without limb dyskinesia, severe early dysphagia, and stridor. There were no exclusion criteria for MSA. Hyposmia, which is commonly associated with COVID-19 (5) and is an exclusion feature for MSA (4), was not present.

Sudden death, reported in this patient, is a relatively specific occurrence in MSA, uncommonly observed in other Parkinsonian syndromes (6). Age at onset was typical for MSA-P, but survival was shorter than average (slightly longer than 2 years). This short duration is however not outside the reported range of MSA progression trajectories. Parkinsonian presentation with predominant urinary dysautonomia, as observed in this patient, is associated with shorter survival (7).

Dopamine transporter (DAT) imaging with TRODAT-1 SPECT cannot distinguish between different Parkinsonian syndromes, including MSA, and cannot differentiate MSA-P from PD and MSA-C (8). However, MSA subtypes have been reported to have characteristic patterns of FDG uptake on PET scan. A typical pattern observed in MSA-P subjects shows diffuse hypometabolism in putamen–pallidum with relative sparing of the caudate nuclei (8); but hypometabolism in the frontal, temporal, parietal, and limbic areas has been observed in MSA patients, particularly among those with reduced MMSE scores (9, 10). In this patient, MMSE scored normal, but an extensive neuropsychological assessment revealed mild executive and visuospatial impairment.

The patient did not consent to perform both the MIBG scan and lumbar puncture. The first could have supported the differential diagnosis between PD and MSA (4, 11), while the search for onconeural and neural surface antibodies could have assessed the hypothesis of immune-mediated or paraneoplastic Parkinsonism.

Reported cases of Parkinsonism following COVID-19 encompass a variety of phenotypes and also include cases of encephalopathy (2). The heterogeneity of reported cases is remarkable and raises the question of whether other MSA cases may have been labeled generically as post-COVID Parkinsonism. Some reports of symmetric akinetic-rigid Parkinsonism without tremors suggest an atypical onset and disease course. Some of these cases may have had MSA or PSP, but their diagnosis may have been overshadowed by the COVID-19 emergency (2, 12). Different mechanisms have been postulated, by which COVID-19 may cause a neurodegenerative condition or accelerate a pre-existing one, but there is currently no robust experimental support. The hypothesized mechanisms include direct CNS invasion, hypoxia and vascular damage, virus-induced cytokine storm, post-infectious immune-mediated events, or the unmasking of prodromal Parkinsonism (13). A variety of movement disorders have been shown to occur or worsen following SARS-CoV-2 infections, including cases with a typical essential tremor phenotype (14).

As for other movement disorders, a chronological relationship between COVID-19 infection and the new onset of Parkinsonism may not necessarily indicate causality. Coincidental occurrence of common medical conditions has been described, for example, PD and multiple sclerosis have been reported to coexist (15). Until now, a few cases of COVID-19-related Parkinsonism have been reported. A causal relationship between SARS-CoV-2 infection and Parkinsonism was not found in any of them, raising the possibility that at least some were coincidental to COVID-19. Of note, this patient tested positive for COVID-19 for ~3 months without respiratory symptoms, suggesting that a systemic inflammatory response to SARS-CoV-2 may have persisted. Hence, this observation raises the question of whether SARS-CoV-2 infection may have accelerated coincidental Parkinsonism. We report a chronological connection with COVID-19 without evidence of a deterministic causative link, notwithstanding the patient, and caregivers retained that Parkinsonism was consequent to COVID-19 infection.

We consider this a case of MSA-P coincidental with COVID-19. Arguably, SARS-CoV-2 infection could have provided an environmental trigger to aggravate the clinical course of the naturally occurring Parkinsonism. Parkinsonian syndromes usually have long prodromal phases when the cardinal features may escape the attention of patients or caregivers (16). In this patient, early motor features, prevalent to the non-dominant side, may have been unnoticed until a virulent SARS-CoV-2 infection raised attention to details of the patient's status. The implementation of the current diagnostic criteria for Parkinsonian syndromes is also supportive to discriminate against coincidental occurrences.

## Data availability statement

The original contributions presented in the study are included in the article/supplementary material, further inquiries can be directed to the corresponding author.

## Ethics statement

Ethical review and approval was not required for the study on human participants in accordance with the local legislation and institutional requirements. The patients/participants provided their written informed consent to participate in this study. Written informed consent was obtained from the participant for the publication of any potentially identifiable images or data included in this article.

## Author contributions

PP collected data and prepared the first draft. AA, AC, EP, and TD collected data and reviewed the manuscript. All authors contributed to the article and approved the submitted version.
